# Self-Induced Core–Shell InAlN Nanorods: Formation and Stability Unraveled by Ab Initio Simulations

**DOI:** 10.1021/acsnanoscienceau.2c00041

**Published:** 2022-10-28

**Authors:** Manoel Alves Machado Filho, Ching-Lien Hsiao, Renato Batista dos Santos, Lars Hultman, Jens Birch, Gueorgui K. Gueorguiev

**Affiliations:** † Thin Film Physics Division, Department of Physics, Chemistry, and Biology (IFM), Linköping University, SE 581 83Linköping, Sweden; ‡ Universidade Maurício de Nassau − UNINASSAU − Unidade Vitória da Conquista, 45020-750Vitória da Conquista, Bahia, Brazil; § Instituto Federal de Educação, Ciência e Tecnologia Baiano, 46880-000Itaberaba, Bahia, Brazil

**Keywords:** self-induced InAlN core−shell nanorods, synthetic growth concept, DFT, reactive magnetron sputter epitaxy, precursor species, nucleation and structural evolution of nanostructures, immiscible systems at nanoscale

## Abstract

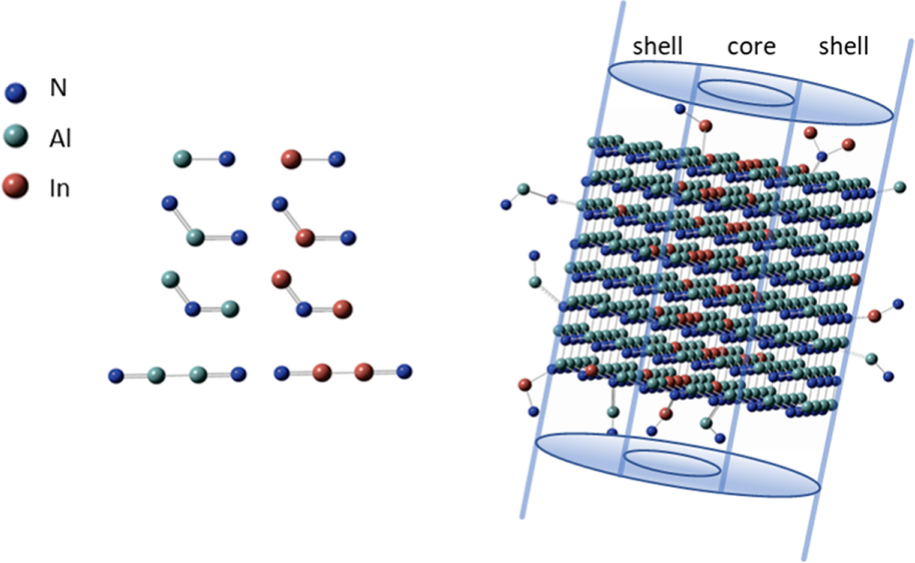

By addressing precursor prevalence and energetics using the DFT-based synthetic growth concept (SGC), the formation mechanism of self-induced InAlN core–shell nanorods (NRs) synthesized by reactive magnetron sputter epitaxy (MSE) is explored. The characteristics of In- and Al-containing precursor species are evaluated considering the thermal conditions at a typical NR growth temperature of around 700 °C. The cohesive and dissociation energies of In-containing precursors are consistently lower than those of their Al-containing counterparts, indicating that In-containing precursors are more weakly bonded and more prone to dissociation. Therefore, In-containing species are expected to exhibit lower abundance in the NR growth environment. At increased growth temperatures, the depletion of In-based precursors is even more pronounced. A distinctive imbalance in the incorporation of Al- and In-containing precursor species (namely, AlN/AlN^+^, AlN_2_/AlN_2_^+^, Al_2_N_2_/Al_2_N_2_^+^, and Al_2_/Al_2_^+^ vs InN/InN^+^, InN_2_/InN_2_^+^, In_2_N_2_/In_2_N_2_^+^, and In_2_/In_2_^+^) is found at the growing edge of the NR side surfaces, which correlates well with the experimentally obtained core–shell structure as well as with the distinctive In-rich core and vice versa for the Al-rich shell. The performed modeling indicates that the formation of the core–shell structure is substantially driven by the precursors’ abundance and their preferential bonding onto the growing edge of the nanoclusters/islands initiated by phase separation from the beginning of the NR growth. The cohesive energies and the band gaps of the NRs show decreasing trends with an increment in the In concentration of the NRs’ core and with an increment in the overall thickness (diameter) of the NRs. These results reveal the energy and electronic reasons behind the limited growth (up to ∼25% of In atoms of all metal atoms, i.e., In_*x*_Al_1–*x*_N, *x* ∼ 0.25) in the NR core and may be qualitatively perceived as a limiting factor for the thickness of the grown NRs (typically <50 nm).

## 1 Introduction

Wurtzite (wz) structure group IIIA nitrides consisting of AlN, GaN, InN, and their alloys unite valuable electronic and engineering properties, including a direct band gap that is tunable in a wide range from near infrared (InN ∼ 0.64 eV) to deep ultraviolet (AlN ∼ 6.2 eV), high thermal stability with high chemical inertness, and high breakdown voltages.^1−6^ These valuable properties enhance the prospects of group IIIA nitrides for applications such as high-brightness light-emitting diodes (LEDs), high-efficiency solar cells, and high electron mobility transistors. However, nitride thin films often generate a high degree of dislocation, which deteriorates the device’s performance. Therefore, one-dimensional (1D) nanostructures are becoming attractive as the dislocations can be easily depleted to the surface.

The growth of binary IIIA nitride nanorods (NRs) has been approached by a diversity of methods, such as metal–organic chemical vapor deposition (MOCVD),^3,7^ molecular beam epitaxy (MBE),^8−13^ and magnetron sputter epitaxy (MSE).^14−17^ With interplaying adatoms’ diffusion and desorption on the growth surface, both self-induced and selective-area-grown NRs can be obtained on various substrates including sapphire, SiC, Si, and metals. Notably, most growth approaches to self-induced NRs retain important methodological advantages such as simplified fabrication not requiring catalysts, advanced lithography, or substrate pre-treatments.^9,10^

Recently, much attention has been paid to ternary IIIA nitrides, including InGaN, InAlN, and AlGaN nanostructures, because their band gaps and lattice parameters can be engineered by tuning their compositions.^4−6^ To obtain high crystal quality, nanostructures are often grown at elevated temperatures. However, instead of forming single-phase ternary alloy NRs, a core–shell structure is found in these self-induced NRs.^7,11−13,18−20^ Presently, most studies addressing formation of self-induced NRs depart from stipulating models relying on a thermodynamical equilibrium viewpoint and phase separation due to the immiscibility of a ternary alloy, which, in general, are not capable of explaining the formation of nanostructures of such complexity and diversity. For instance, AlGaN is miscible throughout the entire compositional range while a core–shell structure is still observed in self-indued NRs.^7^

In the context of the experimental advances of self-induced NRs, part of our team has reported on the core–shell formation in self-induced InAlN NRs by growing them directly onto electron-transparent amorphous carbon (a-C) substrates using reactive MSE.^15^ Scanning transmission electron microscopy (STEM) analysis was employed to address the structural and compositional evolution of core–shell InAlN NRs in their distinct stages of nucleation of discrete In-enriched and Al-rich islands that grow and coalesce during the NR axial–radial growth. The distinctive core–shell structure has been attributed to separate nucleations of Al-rich and In-enriched islands, due to formation of interfaces with diverging interfacial energies and chemical potentials. The In-enriched domains feature a higher growth rate, thus forming the core, while the shell results from the slower growing surrounding Al-rich shell. These observations provide the starting points for the present computational study focused on aspects of NR formation.

Due to the advanced stage of accumulation of experimental data from their growth and characterization, InAlN NRs^15,19−21^ represent an excellent model system to study self-induced NRs in general. Unsolved questions about the formation and stability of core–shell InAlN NRs, including how the very sizable single-phase In_*x*_Al_1–*x*_N immiscibility gap ranging 0.1 < *x* < 0.9^22,23^ impacts NR formation, can the precursor species properties and bonding behavior at the edge of the growing NRs be related to the NR structure and composition, and what the differences are between the energetics of In-rich and In-poor domains and how they influence NR phase stability, remain quantitatively unsolved. Addressing such questions by modeling contributes a better understanding of the interplay between growth conditions and the properties of the as-grown core–shell InAlN NRs and may assist in tuning NR growth conditions, thus achieving grown “on-demand” NRs for a given application.

The synthetic growth concept (SGC) is an extensive approach addressing the growth behaviors of thin films and nanostructures that was employed to successfully elaborate the properties of inherently nanostructured carbon-based materials with potentially varying morphology together with precursors and their role in the formation of nanostructured compounds and architectures in vapor-phase deposition techniques.^24−29^ In this study, we carry out theoretical modeling based on the density functional theory (DFT) employing the SGC to assess and understand the formation of core–shell structures and the stability and electronic properties of InAlN NRs grown by MSE. The following studied key issues are elaborated: (i) the precursor species comprising atoms, dimers, trimers, and tetramers; their stability, abundance, and impact; and their interaction at the edge of the forming NRs; (ii) aspects of growth influencing NR evolution from nucleation to mature and well-defined core–shell NRs; and (iii) dependence of NR electronic properties on the lateral thickness and core composition.

## 2 Methods

Except where explicitly stated, the simulation framework that is adopted in this work is the one of the DFT within its generalized gradient approximation (GGA) at the Perdew–Burke–Ernzerhof (PBE) level of theory as implemented in the Quantum Espresso code.^30,31^ The code employs plane-wave basis sets and projector augmented wave method (PAW) pseudo-potentials. The kinetic energy and the charge cutoffs applied are 100.0 and 600.0 Ry, respectively.

For the purposes of optimization and comparison of precursor species, separate test calculations are carried out by also employing the Gaussian code^32^ at the PBE and PW91 levels of theory.

We employ model systems of different lateral thicknesses, which we designate as thin NRs (324 atoms per supercell) and thick NRs (768 atoms per supercell), both featuring periodic boundary conditions (PBCs) along the growth direction/*c* axis. The NR systems represent a good as possible compromise between presently available computational resources for approaching NRs at the computationally demanding DFT level of theory that we employ in SGC simulations and still being able to meaningfully incorporate in them the core–shell structure of an NR that is integrally inbuilt in their supercells. Thus, in all simulations, the NR models contain distinctive cores and shells with an integrated core–shell interfacial region. Contributing to a more realistic modeling of the NRs, together with the corresponding compositions of the NR core and shell, inbuilt also in the supercell core–shell interface is an implicit feature of the NR models that impacts all reported results, including the results on energy gain at bonding to the NR edge, as well as those on the NR band structure. The presently modeled NRs reach about 60% of the diameters of comparable experimentally grown core–shell InAlN NRs, which generally vary in the range 30–50 nm.^15,19−21^

For the structural relaxations of the model systems representing all core–shell InAlN NRs, their atomic constituents were allowed to relax until the forces decrease to values less than 0.01 eV Å^–1^. The energy convergence criterion in geometry optimizations was 10^–5^ eV. In order to avoid spurious interactions with images of supercells of the NR, a sufficiently large vacuum ring (25 Å) around the NR structures was employed in all relevant calculations.

In the quest for an explanation of the observed stability and of electronic property trends with the variation of the elemental composition of the InAlN alloying in their cores and shells, NRs with varying incorporation rates of In in their cores and shells have been investigated and compared.

When designing both the thin and the thick NR model systems and for a given In concentration in the core, we have studied different distributions of the atomic site positions for the In atoms. For In concentrations in the core below 12–15%, the differences (in cohesive energy and electronic properties) resulting from In atoms at different atomic sites are negligible. For In concentrations above 15–25%, the In-distribution-related cohesive energy differences remain small to statistically insignificant (in any case, below 0.1 eV/at.). The structures that are the most energetically favorable (but by a small margin) tend to be the ones in which the In atoms are as much as possible equally distributed in the NR core. In other words, In clustering in the NR core tends to be unfavorable. Only results corresponding to the structures with the energetically most favorable In atom distribution at a given In concentration in the NR core are reported and discussed.

## 3 Results and Discussion

### 3.1 The Role of Precursors in the Synthetic Growth of Self-Induced Core–Shell InAlN Nanorods

#### 3.1.1 Energetics

**Figure 1:**
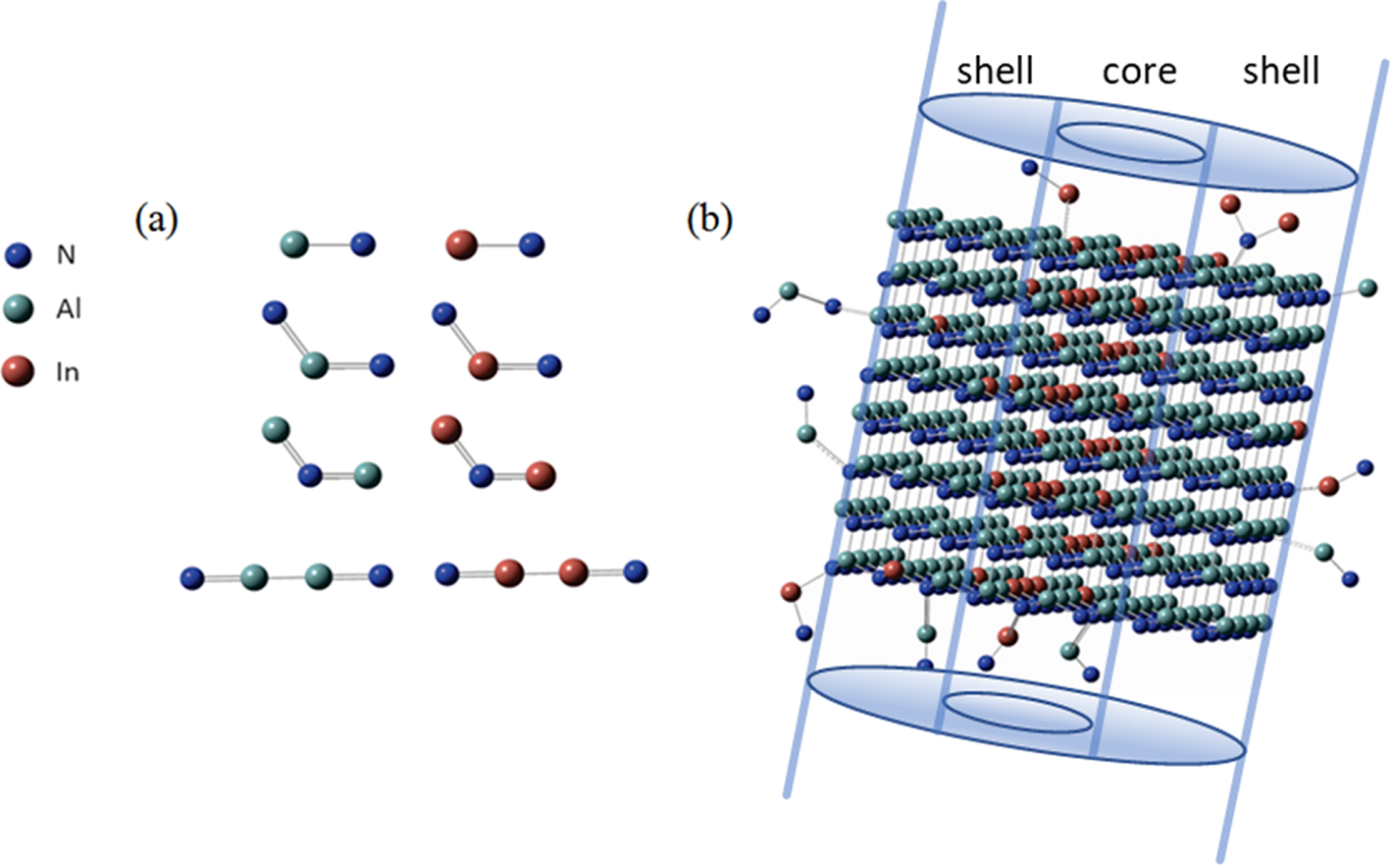
(a) Selection of AlN and InN precursors relevant to the growth of self-induced core–shell InAlN nanorods from MSE synthesis. See the main text for details. (b) AlN and InN precursor species incoming to and bonding at the edge of a well-structured (with clearly delineated core and shell) InAlN NR.

MSE growth of InAlN NRs includes a diversity of neutral and ionized precursor species from co-sputtering of high-purity aluminum and indium targets in a pure nitrogen (N_2_) atmosphere at a working pressure of 5 mTorr and temperatures of 600–700 °C.^15,19−21^ Besides prevalent In and Al single atomic species, a variety of dimers, trimers, and possibly some tetramers need to be considered. As judged by the availability of single Al, In, and N neutral and ionized species, the plausible permutations of (up to four-atomic) precursor species (Figure 1) are investigated for their stability-related characteristics, namely, their cohesive and dissociation energies (Table 1). The calculated cohesive and dissociation energy values are indicative of the precursors’ abundance.

**Table 1:** Stability-Related Characteristics That Are Indicative of Their Abundance, as well as Calculated Energy Gains at Bonding to the Growing Edge of an NR^a^ for Small Precursor Species Relevant to the MSE Growth of Self-Induced Core–Shell InAlN NRs^b^

precursor species	cohesive energy, eV/at.	dissociation energy, eV	energy gain at bonding, eV	precursor species	cohesive energy, eV/at.	dissociation energy, eV	energy gain at bonding, eV
AlN	2.12	4.24	0.27 (0.13)	InN	1.76	3.52	0.15 (0.11)
AlN^+^	1.33	2.66	0.31 (0.13)	InN^+^	1.17	2.34	0.18 (0.12)
AlN_2_	1.59	3.01	0.16 (0.11)	InN_2_	1.39	2.59	0.12 (0.06)
AlN_2_^+^	1.21	2.06	0.22 (0.12)	InN_2_^+^	1.18	1.97	0.16 (0.06)
Al_2_N	1.43	2.67	0.14 (0.06)	In_2_N	1.25	2.39	0.09 (0.04)
Al_2_N^+^	1.07	1.98	0.21 (0.08)	In_2_N^+^	0.97	1.77	0.13 (0.06)
Al_2_N_2_	1.66	1.21	0.11 (0.05)	In_2_N_2_	1.20	0.55	0.04 (0.04)
Al_2_N_2_^+^	1.32	1.07	0.14 (0.06)	In_2_N_2_^+^	0.79	0.63	0.06 (0.04)
Al			0.12 (0.09)	In			0.05 (0.05)
Al^+^			0.19 (0.18)	In^+^			0.09 (0.06)
Al_2_	1.61	3.22	0.08 (0.03)	In_2_	0.77	1.54	0.05 (0.04)
Al_2_^+^	1.15	2.30	0.11 (0.04)	In_2_^+^	0.38	0.76	0.07 (0.06)
N_2_	4.92	9.84		N_2_^+^	4.21	8.42	

a In parentheses are energy gains at bonding at the earlier stages of growth.

b See the main text for details.

The cohesive energy of a precursor species is the energy required for breaking it into isolated atomic species, *E*_coh_ = |*E*_total_ – ∑*E_i_*|, where *i* corresponds to its different constituent atoms. Typical dissociation paths for the species include MN → M + N, MN^+^ → M^+^ + N, MN_2_ → M + N_2_, MN_2_^+^ → M^+^ + N_2_, M_2_N_2_ → M_2_ + N_2_, and M_2_N_2_^+^ → M_2_^+^ + N_2_. Trivially, for all diatomic species, the dissociation energies *E*_dis_ are equal to twice the value of the cohesive energies per atom *E*_coh_ (Table 1).

Neutral and ionized mixed four-atomic species such as Al_2_N_2_ and In_2_N_2_ are the largest species considered. For kinetic reasons (which preclude the formation of larger precursors) and as assessed in previous SGC-addressed magnetron sputtering arrangements,^24−27^ larger than four-atomic precursor species are not included in the studied precursor selection. Even though the four-atomic precursor species are judged to have a low probability of formation, they are included in the simulations for completeness. Since the cohesive energy of small species may benefit from the species size, Al_2_N_2_ and In_2_N_2_ and to a lesser degree their ionized derivatives exhibit about average or in any case not so small cohesive energies. However, Al_2_N_2_ and In_2_N_2_ as well as their ionized derivatives are not expected to play any significant role in the growth process because there are kinetic impediments for their formation. Even when formed, Al_2_N_2_ and In_2_N_2_ and their ionized derivatives easily dissociate into two dimers (Table 1).

Thus, the neutral and ionized mixed dimers and trimers are among the most feasible building blocks of the InAlN NRs. As seen from Table 1, the dimers, once formed, are quite stable (high cohesive energies and high dissociation energies) and what is essential is that the energy gains from their bonding to the growing NR edge are among the highest when all species are compared.

When considering single metal species Al, Al^+^, In, In^+^, and the corresponding homonuclear dimers Al_2_, Al_2_^+^, In_2_, and In_2_^+^, it can be observed that metal dimers are expected to be present in the growth environment due to high cohesive and dissociation energies. However, the gain of their bonding to the growing NR edge is consistently smaller than the gain for the corresponding single metal species, thus exposing the homonuclear dimers as less impactful to the NR growth, which maybe one of several factors contributing to the lack of experimental evidence for any metal atom segregation in core–shell NRs.

The neutral and ionized N_2_ and N_2_^+^ molecules possess high cohesive energies, which reflect their very strong bonding. Consequently, they are not prone to any dissociation in the growth environment of InAlN NRs and at the relevant temperatures.

When evaluating the abundance and the prevalence of all precursors in the deposition environment, usually, their cohesive and the dissociation energies are compared to the thermal energy, which, at ∼700 °C, is ∼0.085 eV, meaning that precursors with both their cohesive and dissociation energies significantly above the threshold of ∼0.09–0.1 eV are expected to prevail in the deposition environment and thus react with a higher probability with the NR growing edge than precursors with energies significantly lower than the thermal energy.

From the data listed in Table 1, it becomes clear that the mixed dimers (both neutral and ionized) and to certain extent also their mixed trimer analogues, as well as the single metallic species, and as compared to thermal energy at the growth conditions exhibit (a) increased thermal stability and (b) increased stability against dissociation. This is an indication of higher abundance/prevalence for these species in the growth environment.

The mixed precursors impact the MSE growth of self-induced core–shell InAlN NRs in the following way: when a mixed dimer or a mixed trimer precursor bonds to the growing edge of an NR, it contributes to the growing structure by a metal atom–nitrogen atom bond; i.e., it imports an Al–N (alternatively an In–N) bond to the edge of the NRs. In comparison to bonding of single atomic species, such a direct import of Al–N/In–N bonds is a particularly efficient contribution to the growth of well-structured InAlN since its basic building blocks (basic unit cell) consist of metal atom–nitrogen atom bonds. As indicated by precursor energetics (Table 1), the precursors’ bonding preferences/gains show an aspect of growth stage dependence; i.e., these preferences are of less importance at the nucleation stage when they bond to a nucleation seed possessing many dangling bonds and being structurally far from the core–shell structure of the NR, while they are of larger importance when the core–shell structure of the NR is already well-formed. It is suggestive, however, that many of the precursors relevant to the growth process (Figure 1a and Table 1) do contain a metal–nitrogen bond—either Al–N or In–N. A similar mechanism of bond import as favoring crystalline order in the growing group IIIA nitride nanostructure is also known from other growth techniques and conditions, e.g., at MOCVD of AlN on epitaxial graphene.^33^ In addition, the cohesive and dissociation energies of In-containing precursors are lower than those of their Al-containing counterparts, indicating that In-containing precursors are more weakly bonded and more prone to dissociation. Therefore, the In-containing species are expected to exhibit somewhat lower abundance in the NR growth environment, which would be even more pronounced at higher growth temperatures.

#### 3.1.2 Impact of the Precursor Species on the Formation of Core–Shell NRs

We pose two hypotheses for the sequence of processes behind NR formation: (i) their core mostly emerges first and then serves as a template for the surrounding growth of their shell and (ii) the core and shell form simultaneously because of surface-initiated spinodal decomposition. While the first hypothesis is not feasibly verifiable by DFT-based simulations—it would require mesoscopic modeling from DFT-obtained parameters—experimental results show nucleation, coalescence, and growth of AlN- and InN-rich islands instead of spinodal decomposition.^23^ Thus, we focus on inquiring the impact of the precursor energetics on the second hypothesis. An essential aspect of understanding the mechanism of NR shell growth is related to the precursor energetics when bonding to (sticking to) the growing NR shell edge. The events of precursors-to-growing-edge bonding were simulated according to the usual protocol as followed in the SGC and is described elsewhere.^28^ The energy gains at bonding of the corresponding precursor species to the NR growing edge should also be compared to the thermal energy at the growth temperature to thus assess the likelihood of thermal desorption of these precursors that might immediately follow their adsorption.

The precursor bonding energetics was investigated in two different situations chronologically corresponding to the two main growth stages: (i) bonding/sticking to irregular InAlN clusters (islands) containing up to tens of atoms, possessing a significant number of dangling bonds, and representing the earlier stages of NR growth and (ii) bonding/sticking to the edge of well-formed NRs with a wurtzite crystal structure containing hundreds of atoms and representing the later stages of NR growth.

Different orientations of each precursor species to the edge of the NR fragment exhibiting (or not) vacancies/dangling bonds were tested, so that the energy gain from the precursor-to-shell bonding is maximized. The energy gain resulting from a precursor species bonding to the NR fragment is expressed as
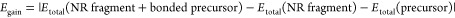


At the first stage of bonding events (i), the energy gains at precursor bonding that vary in the range 0.14–0.03 eV (Table 1, in parentheses in the “energy gain at bonding” column) are relatively low, frequently smaller than the relevant thermal energy of about 0.09 eV, while the values do not show so clear a difference between In- and Al-containing counterparts; i.e., the energy gains at bonding are not particularly metal specific as they happen in stage (ii) of bonding events (see below). These differences can be qualitatively explained with the irregular, cluster-like NR fragments serving as model systems at stage (i). They are rich in dangling bonds. They also best represent the early stages of NR growth when precursors interact with small In- or alternatively Al-rich clusters or islands lacking any well-defined crystals or, for that matter, a wurtzite-like structure.

From comparing bonding events and precursor desorption events (cf. the thermal energy mentioned above) alone, at these early stages of growth, neither In- nor Al-containing species could be clearly identified as bonding proprietarily. Still following hypothesis (ii) for the sequence of processes behind NR formation, the early stages of growth (nucleation) are more relevant to the formation of the cores and of the interior of the shells of the NRs.

When analyzing the picture of the precursor selection as unrelated to bonding events to the NR edge, the SGC results (Table 1, cohesive energy and dissociation energy columns) predict higher abundance of Al-containing species in the MSE growth environment. These values should be looked upon together with the energy gains at precursor bonding results. It is clear that at these earlier stages of NR growth, when neither well-defined NR cores nor well-defined the NR shells are formed, the cohesive and dissociation advantages of the Al-containing versus In-containing species are less influential on growth.

Thus, at the earlier stages of NR growth, their formation progresses around nucleation seeds while still no clearly formed core/shell crystal structure is achieved. Instead, simple clustering/precipitation of available species is occurring, thus downplaying the advantages of Al-containing precursors in terms of cohesive energies and dissociation energies, which equalizes and homogenizes the chances of the In- and Al-containing precursors to bond. The results in Table 1 are also consistent with an early-stage growth standpoint favoring the single metal species (Al/Al^+^, In/In^+^) against the polyatomic precursors. At earlier growth stages, the energy gain (favoring polyatomic species) is less impactful, thus single atomic species bond easier to the edge of the growing NRs. In addition, coalescence/competition/rearrangements between In- and Al-rich islands at early growth stage structural rearrangements further attenuate the impact of the higher abundance of Al-containing polyatomic species over their In-containing counterparts. Thus, the SGC results and their interpretation in the context of the early stages of growth correlate well with the In-rich cores of the NRs.

The second stage of bonding events (ii) corresponds to bonding to the edge of the shell of well-formed NRs with a wurtzite crystal structure (Figure 1b). The energy gains at bonding (Table 1, non-parenthesized values in the “energy gains at bonding” column), vary in the range 0.31–0.05 eV and are consistently and significantly larger than those at stage (i). For most of the Al-containing precursors, these values are larger than the relevant thermal energy of about 0.09 eV, suggesting low probability for immediate thermal desorption from the growing edge. Concurrently, for some of the In-containing precursors, the energy gains come close to (In_2_N, 0.09 eV) or fall below (In_2_N_2_, 0.04 eV; In_2_N_2_^+^, 0.06 eV; In_2_, 0.05 eV; In_2_^+^, 0.07 eV) the thermal energy at the typical MSE growth conditions at 700–800 °C (∼0.09 eV), suggesting that significant desorption of In-containing species from the NR shell edge may be constantly occurring during a typical NR growth run. Summarizing, the energy gains very clearly favor the Al-containing precursors as compared to their In-containing counterparts. These factors, taken together with the precursor’s selection picture (cohesive and dissociation energies), which is independent on the bonding events of growth stages, point to an overwhelming advantage for Al-containing precursors versus those with In. The bonding events at the later stage (ii) correlate with a growth phase when the NRs should have their core/shell structure to a large extent already well-structured. At this stage (ii), any subsequent growth occurs mostly at the shell edge by enlarging the shell diameter and not by any kind of precursors clustering/precipitating/segregating. Moreover, it is also at this stage (ii) that the SGC results most markedly point to In deprivation leading to a reduced In content in the NR shell, as corroborated by scanning transmission electron microscopy energy-dispersive X-ray spectroscopy (STEM-EDX) elemental maps displaying the distribution of Al and In in the NRs as a function of the growth time.^15^ The SGC results also suggest that if the growth temperature is lowered (e.g., to 500–600 °C), which is proportionally translated in lowering of the thermal energy, the In deprivation would be reduced (In-containing species becoming less prone to desorption), leading to an increment of the In content in the NR shell. The opposite thermal regime, i.e., higher growth temperatures in the range of 900 °C, acts in the opposite direction, causing a decreased In content as demonstrated experimentally in ref (21).

By assessing the precursor abundance and selection, these SGC results alone cannot provide a fully quantitative explanation of the specific core–shell morphology as well as the well-defined phase separation originating the core–shell interface of the InAlN NRs. The inherent phase separation is observed at nano- to mesoscopic scale ranging tens of nanometers, translated in core diameters ranging 10–20 nm and external (shell) diameters measuring up to 50 nm, while typical NR axial lengths measure up to 100 nm.^15,19−21^ DFT approaches to modeling the structure and energetics of nanostructures, aperiodic or interfacial systems, including the applied SGC here, usually operating at smaller scales and up to ∼20 nm,^24−27^ cannot quite reach this size scale but via abundance and bonding particulars still show clear trends in the direction of experimentally observed formation of NRs. In a more generalized context, our approach paves the way to unraveling the formation mechanism of self-induced NRs throughout a wide variety of ternary alloys and beyond group IIIA nitrides to other compounds such as InGaAs, InGaP, ZnMgO, and ZrTaB.

### 3.2 Structural and Electronic Properties of Self-Induced Core–Shell InAlN Nanorods

#### 3.2.1 Cohesive Energy Trends with NR Composition and Size

To investigate the dependence of the structural stability and the electronic properties of self-induced core–shell InAlN NRs on their composition (the ratio Al/In in the core and in the shell), we investigated scaled-down model systems of the NRs whereby their core and shell regions are realistically modeled and their wurtzite structure is preserved during geometry relaxation procedures. This matches the strong experimental evidence for the wurtzite crystalline structure of the core–shell NRs as consistently exhibited by selected area electron diffraction (SAED) patterns.^15^

**Figure 2:**
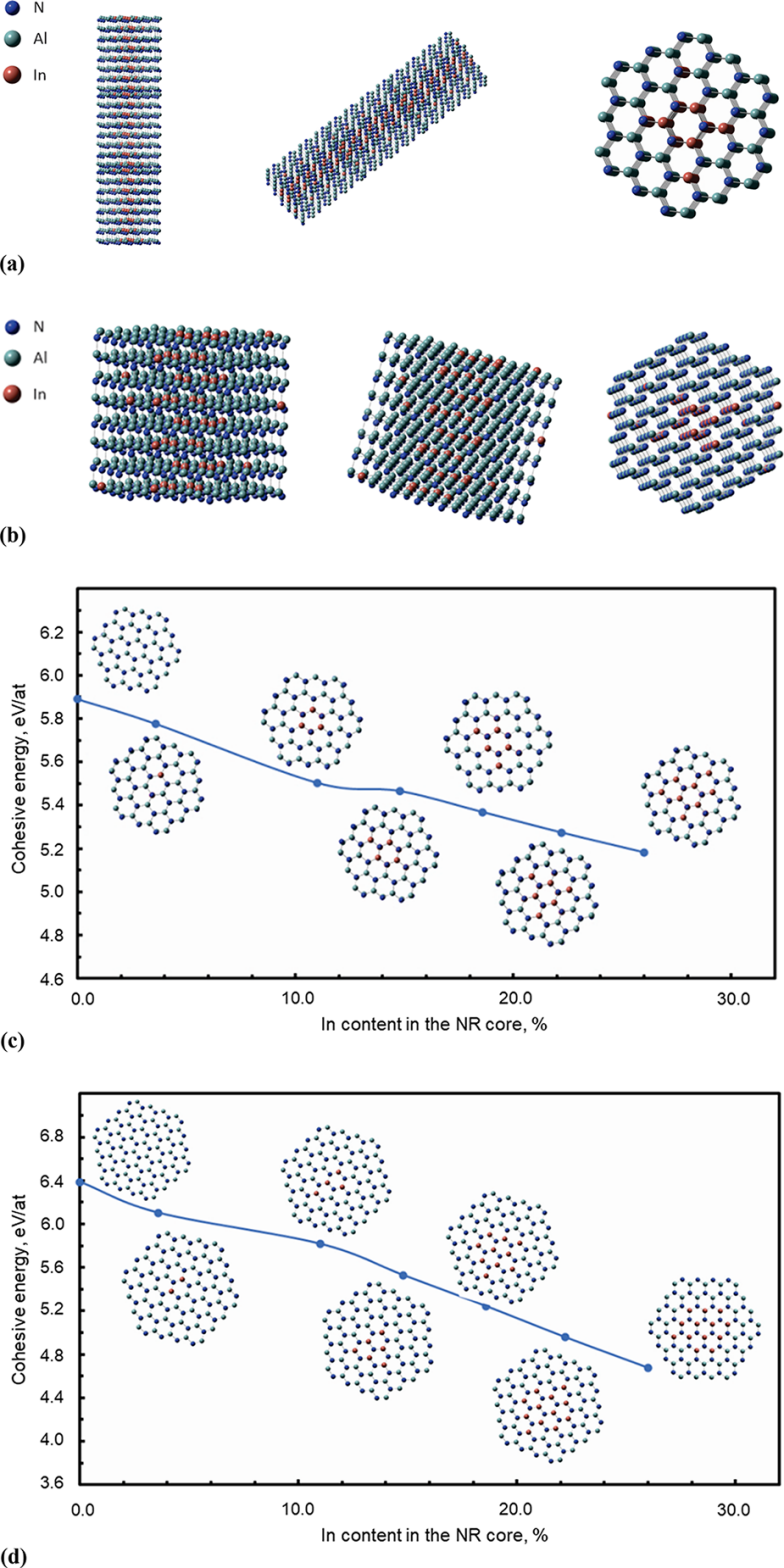
(a) Different representations of the thin NR model system with a diameter of 13.72 nm; (b) different views of a representative of the thick NR model system with a diameter of 17.54 nm. All NR model systems are infinite along the *Z* axis (applying periodic boundary conditions (PBCs)). (c) Cohesive energy per atom as a function of the In concentration in the core for the type of thin NR model system shown in panel (a); cross sections of the NRs with different In concentrations in their core are shown in the corresponding insets. (d) Cohesive energy per atom as a function of the In concentration in the core for the type of thick NR model system shown in panel (b). Cross sections of the NRs with different In concentrations in the core are shown in the corresponding insets.

Figure 2a,b presents different views of two typical core–shell NR model systems with fixed external diameters of 13.72 nm (thin NRs, 324 atoms per unit cell) and 17.54 nm (thick NRs, 768 atoms per unit cell), respectively. It should be kept in mind that both types of model systems are smaller and thinner than experimentally obtained NRs. Thus, keeping as accurately as possible the In-poor shell areas of the NRs the In atoms are quite scarce in the outer regions of both model systems and much visible in their core regions. Figure 2c,d presents the cohesive energies per atom (Table 2) plotted as a function of the In concentration in the core for both thin and thick NRs (see Figure 2a,b, respectively). For clarity, cross sections of the NRs of different In concentrations in the core are shown in the corresponding insets. The evaluation of the cohesive energy at the DFT/PBE level of theory is an important corroboration for the stability of the core–shell NRs. The cohesive energy of the NR model systems is an integral characteristic of the whole nanostructure (comprising its inseparable core and shell) and a measure of its stability. Energy-based criteria alone for the stability of nanostructures are considered indicative but non-conclusive. However, for nanostructures such as the core–shell InAlN NRs that presently are too large to be subjected to realistic ab initio molecular dynamics or to phonon spectra tests but experimentally well-studied and well-documented by a variety of characterization techniques such as TEM and STEM-EDX,^15,19−21^ the energy-based criteria for stability are considered both credible and realistic.^34^

**Table 2:** Band Gaps of Thick and Thin Model Systems of Core–Shell InAlN NRs

	In concentration in the NR core (% of In atoms of all metal atoms)					
NR model system	0	3	11	14	18	22
thin NRs, Figure 2a	0.894	0.886	0.877	0.868	0.842	0.831
thick NRs, Figure 2b	0.848	0.819	0.788	0.761	0.743	0.722

For both the thin and the thick types of model systems, the cohesive energy per atom (obtained for a supercell and then divided by the number of atoms to obtain the cohesive energy per atom, which is more suitable for comparative purposes) decreases with an increasing In concentration in the core. The general similarity and continuity of the energy trends when changing from thin to thick NR models further substantiates the chosen size scaling of the NR model systems and lends credibility when simulation results are extrapolated to the experimentally observed NRs. Notably, the cohesive energy decrement exhibits some deviations for the thin NRs (Figure 2c), while for the thick NRs (Figure 2d), the monotonously decreasing trend is smoother while the cohesive energy per atom is by ∼5–8% lower than for the thin NRs with a comparable In concentration in the core. Consequently, in the case of the highest calculated In content in the core, In_*x*_Al_1–*x*_N (*x* = 0.25), the cohesive energy per atom reads 4.8 eV/at. for the thick NR model system vs 5.2 eV/at. for the thin NR model system. Since the experimentally obtained NRs are often up to twice thicker^15,19−21^ than the thick NR model systems in Figure 2b, extrapolating the present modeling results to the experimentally obtained NRs, their cohesive energy per atom dependence on the In concentration in the core will be one of a monotonous decrement at energies further lowered by ∼5–8%, which suggests for the experimentally obtained NRs with the stoichiometry In_*x*_Al_1–*x*_N (*x* = 0.25) expected cohesive energies per atom of about 4.2 eV. Any NRs with the experimentally observed dimensions and core to shell proportions but with a further increased In content in their core are thus expected to exhibit cohesive energies per atom lower than 4 eV/at. and becoming less and less energetically favorable with any further increment of the In content in their core. This prediction for cohesive energy driving force counteracting In concentrations in the core exceeding 25% in InAlN NRs should be also seen at the background of the cohesive energies per atom for the bulk wurtzite binaries, which, when calculated at the same level of theory, read 7.11 eV/at. for AlN and 5.29 eV/at. for InN.

The energetics of bulk crystals should be taken as a qualitative framework only. Indeed, the energy values for bulk wurtzite AlN and InN should not be expected to be directly comparable to the energetics of NR configurations that have free surfaces and to which PBCs apply only in the growth direction along the *c* axis, ⟨0001⟩. Yet, for lower In concentrations in the core, the cohesive energy graph line corresponding to the thick NR model systems (Figure 2d) indicated higher cohesive energy per atom values of 5.8–6.1 eV/at. compared to those for the thin NR model systems (Figure 2c) of 5.6–5.9 eV/at., while at the opposite end of graphs, i.e., for higher concentrations of In of the core, the thickening of the NRs leads to the opposite trend, and the thick NRs exhibit lower cohesive energies per atom (4.7–5.3 eV/at. (Figure 2d) vs 5.2–5.4 eV/at. (Figure 2c)). Thus, all cohesive energy results corroborate the presumption that thicker NRs are energetically more favorable than thinner ones but up to In concentrations in the NR core of 15–20%; cf. Figure 2c,d. These insights are semi-qualitative: (i) they do not account for growth kinetics at the different growth stages, employing instead well-structured and pre-designed model systems, and (ii) as usual, in DFT-based simulations, the model systems are not subjected to any diffusion processes and concentration adjustments at the core/shell interface or to any concentration modulations. Diffusion processes have been considered to help explain the structure and composition of core–shell nanowires/NRs, which although synthesized by techniques alternative to MSE, namely, Au-catalyzed MBE, are similar to the here studied self-induced InAlN core–shell NRs by featuring Al-rich shells.^35^ The higher Al content in the shells of such MBE-synthesized NRs suppresses the surface diffusivity of other metal atoms such as Ga or In adatoms in the NR sidewalls. This diffusivity-based explanation of the Al-rich shells and Al-poorer cores of a wider class of Al-containing core–shell NRs, although based on different principles from the SGC approach to the structural and compositional evolution of the core–shell NRs envisaged in our present work, reaches similar conclusions regarding the experimentally observed NR compositions.^35^

Geometric relaxation of the NR model systems (Figure 2a,b) was followed by evaluation of their electronic states as well as by performance of the corresponding population analysis thus determining their band gaps (Table 2). The analysis of the band gap dependence on the In concentration in the NR core is considered informative not only because of potential applications but also due to the well-known wide miscibility gap observed in the AlN–InN system and related spinodal decomposition,^36^ which may manifest itself as significant variations of the band gap values of the considered model systems at their relaxations.

**Figure 3:**
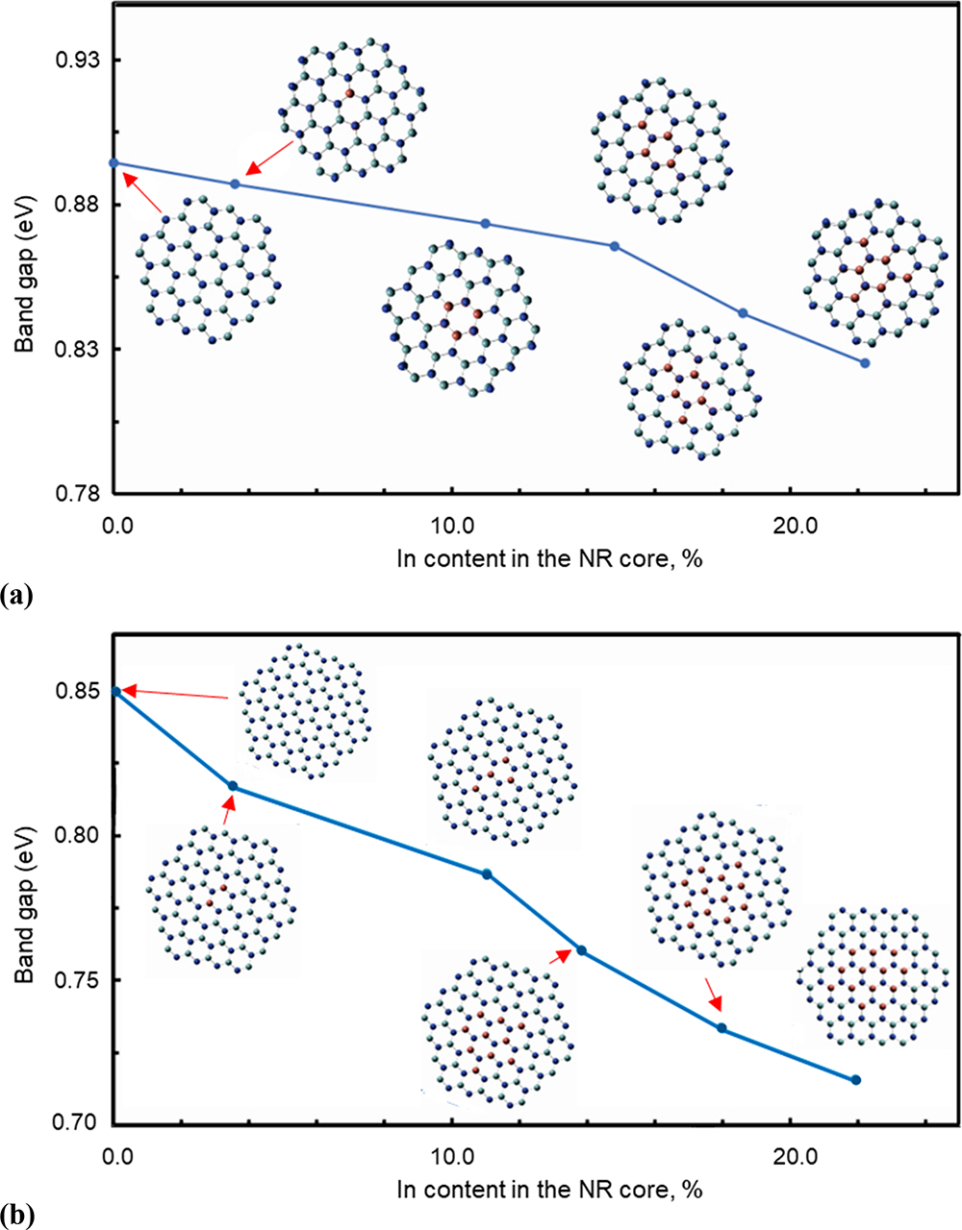
Band gap dependencies on the In concentration in the core of the (a) thin NR model systems shown in Figure 2a and (b) thick NR model systems shown in Figure 2b. For clarity, cross sections of the NRs of different In concentrations in the core are shown in the corresponding insets.

In Figure 3a,b, the band gaps as a function of the In concentration in the core for the thin NR model systems (Figure 2a) and for the thick NR model systems (Figure 2b) are presented. The band gaps are defined here for the inseparable integrally bonded model systems of the NRs without distinguishing between core and shell structural regions or taking into account any possible impact of the core–shell interface. For both types of model systems, the band gap decreases with an increasing In concentration in the core.

As expected, the band gap of the self-induced core–shell InAlN exhibits its highest values (∼0.9 eV) for a pure AlN NRs, while for NRs with a typical core–shell structure and reaching concentrations of above 20% In in their core (with a shell of In concentrations of 2–3%), the band gap has a value of ∼0.83 eV for the thin NR model systems (Figure 2a). A similar band gap trend is found for the thick NR model systems (Figure 2b), in which case for the NRs with a typical, above 20% In content in their core (and 2–3% in the respective shell), the band gap decreases to ∼0.72 eV. Throughout the range of In contents studied, the thickening of the NRs (from the model systems in Figure 2a to those in Figure 2b) leads to a significant decrease in the band gaps of about 7–13%. The decrement of band gaps with the increasing size of simulated nanostructures is a typical size effect due to the increased number of overlapping orbitals as the model systems grow in scale and is observed in a wide range of semiconductor nanomaterials and low-dimensional systems.^37^ An extrapolation similar to the one applied for the NR energetics can also be done for the even thicker experimentally observed NRs, for which band gaps are expected to decrease to values of ∼0.7 eV. Taking into account that the DFT/PBE level of theory significantly underestimates the band gap values of the studied systems but is accurate enough when relative values for revealing trends and tendencies are sought, the obtained band gap range places the core–shell InAlN NRs comfortably in the category of room-temperature semiconductors with all related potential for electronic and optoelectronic applications.

Discussion of the band gaps of inherently non-homogeneously composed materials such as core–shell InAlN NRs poses some non-trivial questions since inhomogeneities at core–shell interfaces are supposed to introduce local band structure disruptions and charge imbalances that may affect the continuity and the concept of a (single) band gap. One aspect of these problematics is how credible the DFT simulations are in this case. In this work, however, the NR model systems employ supercells large enough to incorporate the composition variation/interface and thus assimilate into periodic patterns the inherent inhomogeneity of the NRs for the purpose of PBC calculations. Another conceptually important aspect of the NR band gap is that the theoretically predicted band gap of the entire NRs is mostly defined by their In-richer and therefore tending to lower band gap values core, while the shell’s contribution to the band gaps would be less pronounced. In similarity to quantum well and quantum dot structures, which are also inherently inhomogeneous,^38^ for most practical purposes of potential electronic and optical applications, it is assumed that the measurable band gap of the NRs should match, within the accuracy of the simulations, the predicted band gap values.

The reported results for the cohesive energy and band gap trends taken together suggest that when the core–shell NRs are simulated as thin integral model systems of the wurtzite structure, they do not exhibit any signs of instability observed in bulk systems as related to phase separation issues originating in the wide miscibility gap of InAlN ternaries. If such issues would have occurred during relaxation, they would be detectable as significant structural and morphological changes in the model systems, sharp variations in their cohesive energy dependences on the In content in the NR core (Figure 2c,d), and would impact significantly their band gap trends (Figure 3a,b). Thus, these results point out that bulk immiscibility gaps in core–shell InAlN NRs are mitigated at the nanoscale—a phenomenon facilitated by bonding specificities during their initial nucleation process (as suggested happening for other immiscible systems at the nanoscale^11^). Consequently, immiscibility restrictions appear as efficiently suppressed or non-existent at the nanoscale, being the critical size for that not only above the size of our model systems but possibly also above the size of the experimentally observed NRs as proved experimentally.^15,19−21^

In view of potential electronic and optoelectronic applications of the self-induced core–shell InAlN NRs, it is useful to note that the band gaps of self-induced core–shell NRs are primarily defined by the band structure of their In-richer core. Therefore, by varying the In content in the NR core, the NR band gap can be tuned within at least ∼15% around their DFT/PBE values of ∼0.7–0.8 eV; see Table 2 and Figure 3. The band gap tunability of controllably synthesized and stable at atmospheric conditions nanostructures is frequently perceived as a valuable property, especially considering that the self-induced core–shell NRs are chemically inert nanostructures that are reliably synthesized by a robust deposition method like the reactive MSE.^39,17,19−21^

## 4 Conclusions

We employ DFT-based SGC modeling to acquire knowledge on the formation, stability, and electronic properties of self-induced core–shell InAlN NRs synthesized by reactive MSE. According to the calculated cohesive and dissociation energies of selected precursors, not only single atoms but also dimers, trimers, and their ion counterparts are viable precursors for the growth of InAlN NRs. Further calculations on the energy gains at the bonding of the precursors to the seed and growing edge of the NRs, as well as on the abundance and prevalence-related characteristics of the precursors’ species, are consistently more favorable for Al-containing species as compared to their In-containing analogues, which gives certain advantages to Al incorporation during the whole process of NR growth. The energy gains at the bonding of the precursors to the growing edge of the NRs appear to favor Al-containing precursors only at later stages of NR growth while at earlier stages when the seeds of the core and the core/shell interface are formed, such selection is less pronounced, meaning that In-containing precursors are predicted to be viable building blocks mostly of the NR core. In this context, the SGC results point to In deprivation (as compared to Al) during the thickening of the NR shell at later and concluding stages of the NR growth process, thus corroborating the published experimental evidence for a higher In content in the NR cores.

To further simulate the structural and electronic properties of self-induced core–shell InAlN NRs as dependent on their thickness and the composition of their core, we find that the cohesive energy and the band gaps of the NRs monotonously decrease with an increment of the In content in their core and the thickness of the NR shell. The cohesive energy results can be seen as limiting the growth of NRs that are significantly thicker than the already experimentally observed ones (with diameters not significantly exceeding 50 nm) and of NRs with an In concentration in their core significantly exceeding 25%. The band gap results reveal the self-induced core–shell InAlN NRs as room-temperature semiconductors with band gaps in the range of 0.7–0.8 eV (possibly as low as 0.5 eV depending on their thickness), tunable by varying the In concentration in the NR core.
